# Protein kinase inhibitors substantially improve the physical detection of T-cells with peptide-MHC tetramers

**DOI:** 10.1016/j.jim.2008.09.014

**Published:** 2009-01-01

**Authors:** Anna Lissina, Kristin Ladell, Ania Skowera, Matthew Clement, Emily Edwards, Ruth Seggewiss, Hugo A. van den Berg, Emma Gostick, Kathleen Gallagher, Emma Jones, J. Joseph Melenhorst, Andrew J. Godkin, Mark Peakman, David A. Price, Andrew K. Sewell, Linda Wooldridge

**Affiliations:** a Cardiff University School of Medicine, Heath Park, Cardiff, CF14 4XN, UK; b Department of Immunobiology, King's College London, UK; c NIHR Biomedical Research Centre at Guy's & St Thomas' NHS Foundation Trust and King's College London, UK; d Immune Recovery Section, Med. Klink und Poliklinik II, University of Würzberg, Germany; e Warwick Systems Biology Centre, University of Warwick, Coventry, CV4 7AL, UK; f Haematology Branch, National Heart Lung and Blood Institute, National Institutes of Health, Bethesda, MD 20892, USA

**Keywords:** pMHC, peptide-major histocompatibility complex, TCR, T cell receptor, CTL, cytotoxic T lymphocyte, PBMC, peripheral blood mononuclear cell, PKI, protein kinase inhibitor, CMV, cytomegalovirus, EBV, Epstein-Barr virus, PPI, preproinsulin, T cell, Tetramer, Low avidity CTL, Cancer immunology, Autoimmunity

## Abstract

Flow cytometry with fluorochrome-conjugated peptide-major histocompatibility complex (pMHC) tetramers has transformed the study of antigen-specific T-cells by enabling their visualization, enumeration, phenotypic characterization and isolation from *ex vivo* samples. Here, we demonstrate that the reversible protein kinase inhibitor (PKI) dasatinib improves the staining intensity of human (CD8+ and CD4+) and murine T-cells without concomitant increases in background staining. Dasatinib enhances the capture of cognate pMHC tetramers from solution and produces higher intensity staining at lower pMHC concentrations. Furthermore, dasatinib reduces pMHC tetramer-induced cell death and substantially lowers the T-cell receptor (TCR)/pMHC interaction affinity threshold required for cell staining. Accordingly, dasatinib permits the identification of T-cells with very low affinity TCR/pMHC interactions, such as those that typically predominate in tumour-specific responses and autoimmune conditions that are not amenable to detection by current technology.

## 1 Introduction

T-cells detect antigens in the form of peptides bound to major histocompatibility complex (MHC) molecules at the cell surface. This primary recognition event enables the orchestration of adaptive immunity and targeted destruction of transformed and pathogen-infected cells. T-cell specificity is determined by the highly variable complementarity determining regions of the T cell receptor (TCR). The TCR/peptide-MHC (pMHC) interaction is very weak and classically endures for no longer than a few seconds at physiological temperatures. However, multimerization of pMHC molecules results in cooperative interactivity at the cell surface and ensures that the binding avidity of pMHC tetramers far exceeds the sum of the contributing monomeric affinities (Wooldridge et al., in press). This avidity effect extends the binding half-life of pMHC tetramers (Laugel et al., 2005) and enables stable coherence to the surface of T-cells bearing cognate TCRs (Altman et al., 1996; Burrows et al., 2000). Consequently, pMHC tetramers have transformed the study of antigen-specific T-cells by enabling their visualization, enumeration, phenotypic characterization and isolation from *ex vivo* samples (Altman et al., 1996; Chattopadhyay et al., 2006). Indeed, pMHC tetramers have been used in thousands of studies in the decade since their initial inception and have spawned the formation of several commercial companies.

We have recently used a monoclonal T-cell system to examine T-cell activation and pMHC class I (pMHCI) tetramer binding with a series of altered peptide ligands that vary in their affinity for the cognate TCR by over 100-fold (Laugel et al., 2007). Importantly, cell surface topography, including TCR and CD8 density, remain constant in this system. In this controlled system, efficient staining with tetrameric pMHCI required a monomeric TCR/pMHCI affinity of K_D_ < 35μM; below this threshold, there was a sharp drop off in the intensity of pMHCI tetramer staining (Laugel et al., 2007). A reasonable T-cell agonist in this system bound with a K_D_ ~ 250μM and a weak agonist bound with a K_D_ > 500μM. However, TCR/pMHCI affinities of > 200μM were not detectable by pMHCI tetramer. Thus, using normal staining procedures, pMHC tetramers do not necessarily detect all T-cells that can respond to a particular agonist; similarly, not all agonists for a particular T-cell can be identified physically with pMHC tetramers. These potential limitations of pMHC tetramer staining, which likely extend across a range of multimeric valencies, have important implications for data interpretation and present a particular problem for the detection of tumour-specific or autoreactive T-cells that tend to express low affinity TCRs (Cole et al., 2007).

In this study, we demonstrate that pre-treatment with a protein kinase inhibitor (PKI) enhances pMHC tetramer staining of antigen-specific CD8^+^ and CD4^+^ T-cells and describe the mechanism through which these effects operate. Importantly, these benefits apply only to T-cells that express specific TCRs; PKI treatment does not result in the staining of T-cells that bear non-cognate TCRs. In addition, we show that PKI treatment lowers the TCR/pMHCI affinity threshold required for pMHCI tetramer binding by as much as 5 fold, thereby allowing the binding of pMHCI tetramers to CD8^+^ T-cells that express TCRs with very weak affinities for pMHCI (> 500μM). This simple and universally applicable procedure thereby enables the visualization of previously undetectable tumour-specific and autoreactive CD8^+^ T-cells with pMHCI tetramers through the preferential enhancement of low avidity interactions with TCRs at the cell surface.

## 2 Materials and methods

### 2.1 Cells

The ILA1 CTL clone is specific for the HLA A⁎0201 (HLA A2 from hereon) restricted human telomerase reverse transcriptase (hTERT) epitope ILAKFLHWL (hTERT_540-548_). Mel13 and Mel5 CTL clones are specific for the HLA A2 restricted Melan-A_26-35_ epitope ELAGIGILTV. ILA1, Mel5 and Mel13 CD8^+^ cytotoxic T lymphocyte (CTL) clones were generated and re-stimulated as described previously (Whelan et al., 1999; Laugel et al., 2005). CTL were maintained in RPMI 1640 (Gibco) supplemented with 100 U/ml penicillin (Gibco), 100μg/ml streptomycin (Gibco), 10% heat inactivated fetal calf serum (FCS; Gibco), 2.5% Cellkines (Helvetica Healthcare, Geneva), 200 IU/ml IL-2 and 25 ng/ml IL-15 (Peprotech). CTL lines specific for the influenza matrix protein M1_58-66_ (GILGFVFTL) and Melan-A_26-35_ (ELAGIGILTV) epitopes, both restricted by HLA A2, were generated by pulsing 6 x 10^6^ PBMC from an HLA A2 individual with cognate peptide at concentrations of 1μM and 100μM, respectively, for 1 hour at 37 °C; cells were subsequently washed and resuspended in RPMI 1640 supplemented with 100 U/ml penicillin (Gibco), 100 μg/ml streptomycin (Gibco) and 10% heat inactivated FCS (Gibco) only. After 3 days, increasing amounts of IL-2 were added to the media reaching a maximum concentration of 20 IU/ml by day 14; lines were then tested by pMHCI tetramer staining. Patient samples were collected by leukapheresis; mononuclear cells were isolated by standard Ficoll-Hypaque density gradient centrifugation and stored by cryopreservation. For autoimmune studies, blood was obtained from two HLA A2 patients with type 1 diabetes; both were adults, aged 27 and 31 years, and were studied within 3 months of diagnosis. Short-term lines from these diabetic patients were established as described above using the PPI_15-24_ autoantigen preproinsulin peptide (ALWGPDPAAA) (Skowera et al., 2008); this peptide binds HLA A2 with high affinity (Arif et al., 2004). Naïve murine CTL were obtained by harvesting splenocytes from transgenic F5 Rag^+^ mice. A significant percentage of CD8^+^ T-cells within the splenic populations of these mice express the F5 TCR, which recognizes the H-2D^b^-restricted influenza H17-derived nucleoprotein peptide epitope (ASNENMDAM) (Mamalaki et al., 1993). The HLA DR⁎0101-restricted CD4^+^ T-cell clone C6 recognizes the influenza virus A HA_307-319_ epitope (PKYVKQNTLKLAT).

### 2.2 Protein kinase inhibitors

Dasatinib was synthesized as described previously (Lombardo et al., 2004). Biological activity was tested in a cell death titration assay on BA/F3 bcr-abl cells as described previously (Magnusson et al., 2002). Dasatinib was dissolved in DMSO to a concentration of 1 mM and stored in aliquots at − 20 °C. Once thawed, these stocks of dasatinib were stored at 4 °C and used within 7 days. The 1 mM DMSO stock was diluted 1/10,000 in PBS on the day of experimentation to achieve a working solution of 100 nM; subsequent 1/2 dilution yielded a final concentration of 50 nM in cellular assays unless stated otherwise. The 100 nM working solution was always made up on the day of experimentation as the shelf-life of this solution is short (~ days). Staurosporine (Biomol), Lck inhibitor II (Calbiochem), genestein (calbiochem), herbimycin A (Calbiochem), PP2 (Calbiochem) and PP3 (Calbiochem) were dissolved and stored at − 20 °C in DMSO. PKIs were dissolved in PBS prior to use and tested at concentrations of 1 nM, 3 nM, 5 nM, 10 nM, 20 nM, 50 nM, 100 nM, 250 nM, 500 nM and 1μM.

### 2.3 pMHCI tetramer manufacture

Soluble biotinylated pMHCI monomers were produced as described previously (Wooldridge et al., 2005). Tetrameric pMHCI reagents were constructed by the addition of either R-Phycoerythrin (PE)-conjugated streptavidin (Molecular Probes, Invitrogen) or Alexa 488-conjugated streptavidin (Molecular Probes, Invitrogen) at a pMHCI:streptavidin molar ratio of 4:1. Conjugated streptavidin was added to a solution of soluble pMHCI in 5 equal aliquots at 20 min intervals and subsequently stored in the dark at 4 °C.

### 2.4 pMHCI tetramer staining and flow cytometry: clones and splenoctyes

10^5^ Mel13, Mel5 or ILA1 CTL were pre-treated at 37 °C with dasatinib at a range of concentrations (0–50 nM) for a series of durations up to 1 h. Mel13/Mel5 or ILA1 were then stained with either PE-conjugated HLA A2/ELAGIGILTV or HLA A2/ILAKFLHWL tetramer, respectively, at a final concentration of 10μg/ml for 20 min at 37 °C. The HLA DR⁎0101-restricted clone C6 was stained with HLA DR⁎0101/PKYVKQNTLKLAT PE tetramer for 20 min at 37 °C. After initial experiments to determine the optimal conditions of use, all subsequent experiments were performed by incubating T-cells ± 50 nM dasatinib for 30 min at 37 °C prior to tetramer staining. Unless otherwise stated dasatinib remained present throughout the staining procedure. Subsequent to tetramer staining, CTL clones were stained with anti-human CD8-FITC (clone SK1; BD Pharmingen) and 7-AAD (Viaprobe; BD Pharmingen) for 30 min on ice then washed twice with phosphate buffered saline (PBS); the HLA DR⁎0101-restricted clone C6 was stained with 7-AAD (Viaprobe; BD Pharmingen) only. For murine CTL, 5 x 10^5^ splenocytes were pre-treated with 50 nM dasatinib for 30 min at 37 °C, stained with H2-D^b^/ASNENMDAM PE-conjugated tetramer for 20 min at 37 °C and then anti-murine CD8-Cy5.5 for 30 min on ice, washed twice and re-suspended in PBS. Data were acquired using a FACSCalibur flow cytometer (BD) and analyzed using FlowJo software (Treestar Inc., Ashland, OR, USA).

### 2.5 pMHCI tetramer staining and flow cytometry: human peripheral blood mononuclear cells

Frozen peripheral blood mononuclear cells (PBMCs) were thawed in a 37 °C water bath until a small clump of ice remained and then transferred into RPMI medium containing 10% FCS, 100 U/ml penicillin, 100 μg/ml streptomycin, 2 mM L-glutamine (Gibco) and 100 U DNase/ml (Roche Diagnostics Corporation, Indianapolis, IN, USA). PBMC were washed twice in this medium and then left to rest for 2 h at 37 °C. After 2 washes with PBS, 2 × 10^6^ PBMC were stained with live/dead^®^ fixable violet amine reactive dye (Invitrogen Corporation, Carlsbad, California, USA), washed and incubated for 30 min at 37 °C in PBS alone or PBS containing 50 nM dasatinib. Subsequently, PBMC in 50μl of PBS alone or PBS containing 50 nM dasatinib were stained for 20 min with pHLA A2 tetramers refolded around either CMV pp65_495-503_ (NLVPMVATV), EBV BMLFI_259-267_ (GLCTLVAML) or Melan-A/Mart-1_26-35_ (ELAGIGILTV) peptides. After 2 washes in PBS containing 1% FCS and 0.02% sodium azide (Sigma-Aldrich, St. Louis, MO, USA), cells were stained with a selection of the following cell surface monoclonal antibodies (mAbs): (i) anti-CD3-APC-Cy7 and anti-CD8-APC (BD Biosciences, San Jose, CA, USA); (ii) anti-CD4-PE-Cy5.5 (Caltag Laboratories, purchased through Invitogen Corporation, Carlsbad, California, USA); and, (iii) anti-CD8-quantum dot (QD)705, anti- CD14-Pacific Blue and anti-CD19-Pacific Blue, conjugated in-house according to standard protocols (*http://drmr.com/abcon/index.html*). The latter two mAbs were used to exclude CD14^+^ monocytes and CD19^+^ B cells, which can bind tetramer non-specifically, from the analysis. Finally, cells were washed and resuspended in PBS containing 1% paraformaledehyde (PFA). Stained PBMC were acquired on a BD LSR II (Becton Dickinson Immunocytometry Systems, San Jose, CA, USA) and analyzed using FlowJo software (Tree Star, Inc., Ashland, OR, USA).

### 2.6 TCR downregulation and tetramer on-rate experiments

HLA A2-expressing C1R B cells (Hutchinson et al., 2003) were either pulsed with ELAGIGILTV peptide at a concentration of 10^–6^ M for 60 min at 37 °C or incubated in medium alone. After two washes with RPMI 1640 supplemented with 100 IU/ml penicillin and 100μg/ml streptomycin, 60,000 HLA A2^+^ C1R cells (pulsed or unpulsed) were incubated for 4 h at 37 °C with 30,000 Mel13 CTL that had been pre-treated with PBS ± 50 nM dasatinib for 30 min at 37 °C. Cells were then stained with anti-TCR-FITC (clone BMA 031; Serotec) and anti-CD8-APC (clone RPA-T8; BD Pharmingen) for 30 min on ice, washed twice and resuspended in PBS. Data were acquired using a FACSCalibur flow cytometer and analysed using FlowJo software (Treestar Inc., Ashland, OR, USA). Tetramer on-rate experiments were performed as previously described (Laugel et al., 2007).

### 2.7 Fluorescence microscopy

10^5^ ILA1 CTL were treated with PBS ± 50 nM dasatinib for 30 min at 37 °C, then stained with Alexa 488-conjugated (Molecular Probes) HLA A2/ILAKFLHWL tetramer at a final concentration of 20μg/ml for 15 min at 37 °C. Following two washes with PBS, each sample was fixed in 2% paraformaldehyde. After fixing, ILA1 CTL were re-suspended in 100μl of 2% FCS/PBS and then spun on to a microscope slide at 550 rpm for 5 min using a cytospin. Samples were subsequently analysed on a Leica DM LB2 (Leica Microsystems) fluorescence microscope.

### 2.8 IFNγ ELISpot assays

CD8^+^ T-cell responses to islet autoantigen were detected by IFNγ ELISpot as described previously (Chang et al., 2003) with the following modifications. PBMCs were pre-cultured at 37 °C/5% CO_2_ in single wells of 48-well plates at a density of 1 × 10^6^ cells in 0.5 ml TC medium (RPMI 1640 supplemented with antibiotics (Invitrogen) and 10% human AB serum (PAA, Somerset, UK)) containing the test peptide at a final concentration of 10μM. Control wells contained TC medium with an equivalent concentration of diluent (DMSO). After 24 h incubation, non-adherent cells were re-suspended using pre-warmed TC medium (2% AB serum), washed, brought to a concentration of 10^6^ cells/300μl, and then dispensed in 100μl aliquots into wells of 96-well ELISpot plates (Nunc Maxisorp; Merck Ltd., Poole, UK) pre-blocked with 1% bovine serum albumin in PBS and pre-coated with monoclonal anti-IFNγ (U-Cytech, Utrecht, NL). Assays were then developed according to the manufacturer's instructions; plates were dried and spots were counted using a BioReader 3000 (BioSys, Karben, Germany) and reported as total responder cells per 10^6^ PBMCs.

### 2.9 Intracellular cytokine staining assays

10^6^ CTL were stimulated with specific peptide at a concentration of 10μg/ml for 6 h; brefeldin A (10μg/ml; Sigma-Aldrich) was added for the final 5 h. Unrelated peptide (10μg/ml) was used as a negative control. Briefly, the cells were fixed in paraformaldehyde (2%; Sigma-Aldrich), permeabilized with saponin (0.5%; Sigma-Aldrich), and labeled with APC-conjugated anti-TNFα mAb (BD Pharmingen). The cells were evaluated using a FACSCalibur flow cytometer (BD Biosciences). At least 10,000 events gated on forward and side scatter were analyzed using FlowJo software (Tree Star, Inc., Ashland, OR, USA). Corresponding isotype control antibodies were used to establish the quadrants for analysis.

### 2.10 Mathematical modelling of the dasatinib effect on pMHCI tetramer staining

A mathematical model that relates staining intensity to tetramer binding kinetics has been described previously (Laugel et al., 2007; van den Berg et al., 2007). Briefly, in this model, the high binding avidity of pMHCI tetramers is accounted for by assuming that tetramers can engage up to three TCR molecules, forming a cluster of one, two, or three TCRs in which association and dissociation between the TCRs and the tetramer pMHCI sites occurs at a much higher rate than diffusive escape of temporarily unbound TCRs, tending to stabilize the cluster until the tetramer happens to become disassociated with TCR molecules at all four sites. Moreover, in singlet and duplet clusters, a free TCR can associate contacts with free pMHCI sites to form, respectively, a duplet or triplet cluster. The dasatinib effect is incorporated into this model by assuming that dasatinib alters the rate at which singlet and duplet clusters recruit free TCRs. Thus, the expression for the relative staining intensity I becomes:

$$I=I_{\min}+\Delta_{I}\left(\left(K_{1}/K_{D}\right)r_{0}+\delta_{D}{\left(K_{2}/K_{D}\right)}^{3}r_{0}^{2}+\delta_{T}^{2}{\left(K_{3}/K_{D}\right)}^{6}r_{0}^{3}\right)\text{.}$$

where K_D_ is the single-site dissociation constant; K_1_, K_2_ and K_3_ are kinetic parameters; I_min_ and Δ_I_ relate the read-out to the number of surface bound tetramers (I_min_ is a nuisance parameter, representing the background level); δ_*D*_ is the duplet recruitment enhancement factor and δ_*T*_ is the triplet recruitment enhancement factor. In the absence of dasatinib, we have δ_*D*_ = δ_*T*_ = 1, whereas these factors are greater than 1 if dasatinib promotes TCR recruitment. The scaled free TCR density, *r*_0_, is implicitly defined by the scaled conservation law:

$$1=r_{0}+\left(K_{1}/K_{D}\right)r_{0}+2\delta_{D}{\left(K_{2}/K_{D}\right)}^{3}r_{0}^{2}+3\delta_{T}^{2}{\left(K_{3}/K_{D}\right)}^{6}r_{0}^{3}$$

(from Laugel et al., 2007). Association kinetics has been found empirically to be described very well by the biphasic exponential model:

$$I\left(t\right)=I_{\min}+I_{\max,\;\text{fast}}\left(1-\exp\left(\lambda_{\text{fast}}t\right)\right)+I_{\max,\;\text{slow}}\left(1-\exp\left(\lambda_{\text{slow}}t\right)\right)$$

where *I*(*t*) is the staining intensity at time *t* and *I*_max, fast_, I_max, slow_, *λ*_fast_, and *λ*_slow_ are positive parameters (van den Berg et al., 2007). The goodness of fit is fair but not perfect (Fig. 2B); a better curve fit might be achieved with a more sophisticated model. However, we have taken a minimalistic approach to modelling the dasatinib effect because of the mechanistic uncertainties surrounding its mode of action. The duplet recruitment enhancement factor δ_*D*_ was estimated to equal 6.53 ± 2.93, whereas the triplet recruitment enhancement factor δ_T_ was estimated to equal 13.7 ± 7.38 (by simultaneous non-linear least- squares). The fit of the model indicates that the data are consistent with the hypothesis that dasatinib makes free TCRs more readily available to pMHCI tetramers for recruitment into duplet and triplet clusters.

## 3 Results

### 3.1 PKI treatment enhances pMHC tetramer staining of CD8^+^ and CD4^+^ T-cells

**Fig. 1:**
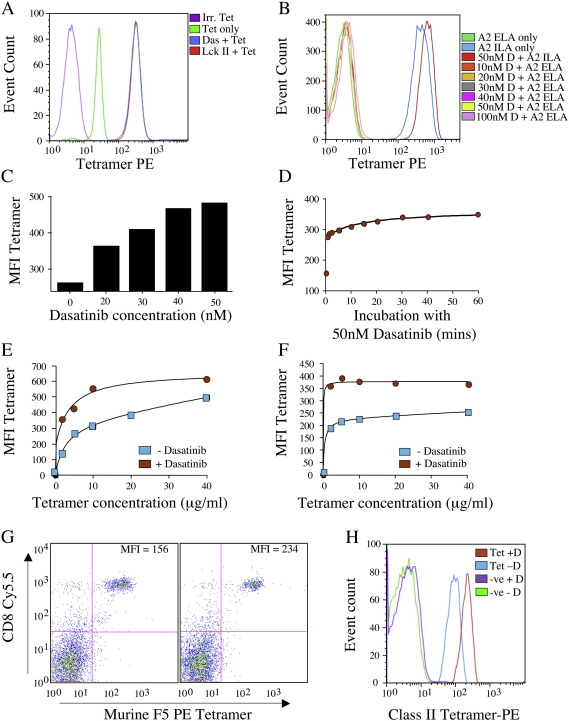
Dasatinib substantially improves pMHC tetramer staining intensity. A. 10^5^ ILA1 CTL were re-suspended in 40μl of PBS ± 50 nM dasatinib or Lck inhibitor II (Calbiochem), then incubated at 37 °C for 30 min. Cells were then stained with cognate HLA A2/ILAKFLHWL-PE tetramer at a final concentration of 10µg/ml for 20 min at 37 °C, washed twice in PBS and analyzed on a FACSCalibur (BD) flow cytometer. A > 10-fold increase in median fluorescence intensity (MFI) was observed after treatment with 50 nM dasatinib (blue) or LcK inhibitor II (red) compared to staining without PKI pre-treatment (green line). B. 10^5^ ILA1 CTL were treated with various concentrations of dasatinib for 30 min at 37 °C, then stained with either HLA A2/ILAKFLHWL tetramer or the non-cognate HLA A2/ELAGIGILTV tetramer for 20 min at 37 °C before washing with PBS. C. 10^5^ ILA1 CTL were resuspended in 40μl of PBS ± the indicated concentration of dasatinib and incubated for 60 min at 37 °C. Cells were then stained with cognate HLA A2/ILAKFLHWL-PE tetramer at a final concentration of 10 μg/ml for 20 min at 37 °C and washed twice in PBS prior to flow cytometric analysis. D. As (A), but ILA1 CTL were incubated with 50 nM dasatinib for various times prior to staining with pMHCI tetramer. For this experiment the drug was washed off prior to staining. E. As (A), but tetramer concentration was varied to stain CTL pre-treated ± 50 nM dasatinib for 30 min. F. 10^5^ Mel13 CTL were stained with various concentrations of HLA A2/ELAGIGILTV tetramer following incubation ± 50 nM dasatinib for 30 min. G. 5x10^5^ splenocytes from an F5 TCR transgenic Rag^+^ mouse were resuspended in PBS ± 50 nM dasatinib and incubated for 30 min at 37 °C. Cells were subsequently stained with H2-D^b^/ASNENMDAM-PE tetramer for 20 min at 37 °C followed by anti-CD8 Cy5.5 for 30 min on ice prior to two washes in PBS and analysis by flow cytometry. H. 10^5^ cells of the HLA DR⁎0101-restricted, influenza virus A HA_307-319_ PKYVKQNTLKLAT-specific CD4^+^ clone C6 were incubated with PBS ± 50 nM dasatinib for 30 min at 37 °C, then stained with cognate PE-conjugated tetramer for 20 min at 37 °C. Samples were washed with PBS before flow cytometric analysis. Irrelevant tetramer was used as a negative control in all cases.

Incubation of ILA1, a CTL clone specific for the HLA A2-restricted epitope hTERT_540-548_ (ILAKFHWL), with the PKIs dasatinib or 3-(2-(1H-benzo[d]imidazol-1-yl)-6-(2-morpholinoethoxy)pyrimidin-4-ylamino)-4-methylphenol (Lck inhibitor II; Calbiochem) resulted in a > 10 fold increase in pMHCI tetramer staining intensity (Fig. 1A). Pre-incubation with the PKI PP2 (Calbiochem) resulted in a moderate increase in tetramer staining; however, no significant enhancement was observed when CTL were pre-treated with herbimycin, PP3, genestein (Calbiochem) or staurosporine (Biomol) (data not shown). Importantly, PKI inhibitor treatment did not enhance staining with non-cognate pMHCI tetramer (Fig. 1B). Identical results were obtained for the HLA A2-restricted Melan A_26-35_ ELAGIGILTV-specific CTL clone Mel13 (data not shown).

Dasatinib is an ATP-competitive, dual Src/Bcr-Abl kinase inhibitor that has recently entered the clinic for the treatment of chronic myelogenous leukaemia (CML) (Shah et al., 2004). In addition, recent data shows that dasatinib can inhibit wild type and mutant forms of KIT, a class III receptor tyrosine kinase. As a result, dasatinib may also have a role to play in diseases associated with KIT activation loop mutations such as systemic mastiocytosis, acute myelogenous leukaemia (AML), seminoma, gastrointestinal stromal tumours and anal melanomas (Schittenhelm et al., 2006; Antonescu et al., 2007). Dasatinib has been shown to be a potent inhibitor of the Src protein kinase Lck (IC_50_ = 0.4 nM) (Carter et al., 2005; Weichsel et al., 2008). Furthermore, while dasatinib reversibly inhibits antigen-specific T-cell effector functions, it is not toxic to T-cells in the short term at concentrations < 100 nM (Weichsel et al., 2008). Indeed, T-cell clones incubated in 50 nM dasatinib for 24 h were able to regain responsiveness to antigen within 1 h of drug removal (data not shown). Dasatinib can also be used in flow cytometry-based applications without loss of cell viability (Weichsel et al., 2008). These properties prompted us to select dasatinib for further investigation in the current study.

The enhancement of pMHCI tetramer staining following dasatinib treatment was highly dose dependent (Fig. 1C). Maximal effect was achieved by exposing CTL to 50 nM dasatinib for 1 h, which resulted in an 89% increase in pMHCI tetramer staining intensity (Fig. 1C). Unexpectedly, pre-incubation of CTL with 50 nM dasatinib for as little as 30 s resulted in a 60% increase in pMHCI tetramer staining (Fig. 1D). Furthermore, incubation with 50 nM dasatinib for 30 min significantly enhanced the staining of both ILA1 and Mel13 CTL over a wide range of pMHCI tetramer concentrations (Fig. 1E&F). Pre-incubation with dasatinib also enhanced pMHCI tetramer staining of naïve murine F5 TCR CTL directly *ex vivo* (Fig. 1G) and pMHCII tetramer staining of a HLA DR⁎010-restricted CD4^+^ T-cell clone (Fig. 1H). Thus, pre-incubation with 50 nM dasatinib for 30 min provides a quick and easy way to enhance pMHC tetramer staining efficiency in both human (CD4^+^ and CD8^+^ T-cells) and murine systems. These effects are highly specific; increased pMHC tetramer binding only occurs in the presence of a cognate TCR/pMHC interaction (Fig. 1B&H).

### 3.2 Dasatinib preferentially enhances pMHCI staining of T-cells bearing low affinity TCRs

**Fig. 2:**
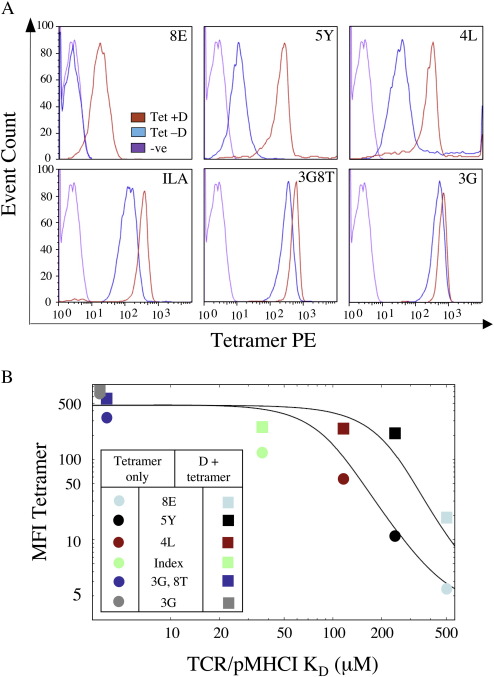
Dasatinib treatment preferentially increases the ability of pMHCI tetramers to stain T-cells bearing low affinity TCRs. A. 10^5^ ILA1 CTL were stained with 10μg/ml PE-conjugated HLA A2 tetramer folded around the 8E, 5Y, 4L, index (ILAKFLHWL), 3G8T or 3G peptides for 20 min at 37 °C following incubation ± 50 nM dasatinib for 30 min at 37 °C. For all samples, data were acquired with a FACSCalibur flow cytometer (BD) and analyzed using FlowJo software. Irrelevant tetramer was used as a negative control. B. The MFI of tetramer staining for all of the variants in the presence and absence of dasatinib displayed in (A) are plotted against the monomeric affinity of TCR/pMHCI interactions previously measured for each of these variants expressed as the dissociation constant (K_D_) (Table 1). Curves were fitted as described in the Materials and methods.

In order to dissect further the effects of dasatinib, we examined pMHCI tetramer staining using several altered peptide ligands for the ILA1 CTL clone that differ in their binding affinity for the ILA1 TCR by > 100-fold (Table 1). Pre-incubation with dasatinib enhanced staining efficiency with all variant pMHCI tetramers (Fig. 2A). The percentage increase in tetramer staining afforded by pre-incubation with dasatinib for 8E, 5Y, 4L, ILA index, 3G8T and 3G pMHCI tetramers was 675%, 1825%, 324%, 111%, 75% and 26%, respectively. Thus, the benefits of dasatinib pre-treatment in terms of enhanced tetramer staining intensity are greater for peptide variants that exhibit weaker interactions with the ILA1 TCR. The intensity of pMHCI tetramer staining in the presence and absence of dasatinib treatment was plotted against the monomeric TCR/pMHCI dissociation constants and a curve fitted according to the mathematical model outlined in the Materials and methods (Fig. 2B). The data demonstrate that, in the absence of dasatinib, there is a sharp reduction in tetramer staining intensity for ligands with TCR/pMHCI K_D_ > 35μM; this is consistent with previous observations (Laugel et al., 2007). In the presence of dasatinib, however, the TCR/pMHCI affinity threshold for this sharp drop-off did not occur until the KD exceeded 200μM. In fact, dasatinib treatment allows detectable staining of the ILA1 clone even when the agonist TCR/pMHCI K_D_ exceeds 500μM. Dasatinib treatment therefore enables the physical detection of CTL bearing TCRs with low affinity for the cognate pMHCI ligand that would otherwise be undetectable using pMHCI tetramer staining alone.

### 3.3 Dasatinib reduces pMHCI tetramer-induced cell death

**Fig. 3:**
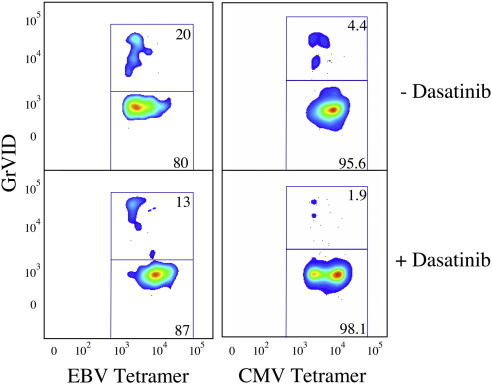
Dasatinib reduces pMHCI tetramer-induced cell death. Increased tetramer staining in the presence of dasatinib appears to be due partly to reduced cell death. It is known that pMHCI tetramer-induced signaling can trigger cell death (Purbhoo et al., 2001; Xu et al., 2001; Guillaume et al., 2003; Cebecauer et al., 2005). Cell death induced by tetramer staining was assessed using the amine-reactive viability dye GrViD at the end of the staining procedure, which is spectrally distinct from ViViD. PBMC were stained with ViViD to identify and allow exclusion of dead and dying cells prior to the addition of pMHCI tetramer; GrVID staining was performed after pMHCI tetramer and surface antibody staining. Data were acquired on a BD LSR II flow cytometer and analyzed using FlowJo software. ViViD^+^, CD14^+^ and CD19^+^ cells were excluded from the analysis and the frequency of GrViD-positive cells was assessed in the tetramer-positive CD3^+^CD8^+^ T-cell populations. Representative flow profiles are shown here for CD8^+^ T-cells specific for the HLA A2-restricted epitopes CMV pp65_495-503_ (NLVPMVATV) and EBV BMLFI_259-267_ (GLCTLVAML). The frequencies of dead cells varied depending on the tetramer used, but the frequency of GrViD-positive dead cells within the tetramer-positive population was always lower in the presence of 50 nM dasatinib. These data, together with comparable results in other systems (data not shown), suggest that the cumulative cell death over the time course of a staining experiment could be substantially reduced by treatment with dasatinib.

Previous studies have shown that soluble pMHCI tetramer-induced signaling can trigger cell death (Purbhoo et al., 2001; Xu et al., 2001; Guillaume et al., 2003; Cebecauer et al., 2005). This can reduce the number of live cells that remain after pMHCI tetramer staining under normal conditions. Dasatinib blocks antigen-specific signaling and subsequent T-cell effector functions (Weichsel et al., 2008). Consequently, we hypothesized that dasatinib could prevent pMHCI tetramer-induced cell death. Indeed, the percentage of tetramer-positive cells that died when PBMCs were stained directly *ex vivo* with pMHCI tetramers representing epitopes derived from cytomegalovirus (CMV) and Epstein-Barr virus (EBV) was reduced in the presence of dasatinib (Fig. 3). Therefore, dasatinib exerts three beneficial effects: (i) it increases the intensity of pMHCI and pMHCII tetramer staining; (ii) it preferentially enhances pMHCI tetramer staining of T-cells bearing low affinity TCRs; and, (iii) it reduces pMHCI tetramer-induced cell death.

### 3.4 Substantial improvements in the detection of antigen-specific CD8^+^ T-cells directly ex vivo

**Fig. 4:**
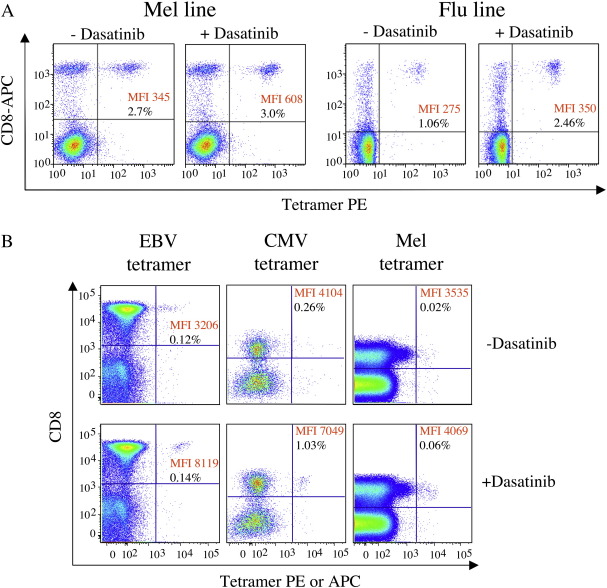
Dasatinib enhances the visualization of antigen-specific CD8^+^ T-cells in mixed cell populations. A. Staining of HLA A2-restricted CTL lines expanded from PBMC by one round of stimulation with the influenza matrix M1_58-66_ peptide (GILGFVFTL) or the Melan-A/Mart-1_26-35_ peptide (ELAGIGILTV). Lines were stained with cognate tetramer ± pre-treatment with 50 nM dasatinib for 30 min at 37 °C. B. Flow cytometric profiles of live CD3^+^ lymphocytes stained with HLA A2 tetramers folded around the EBV BMLF1_259-267_ (GLCTLVAML), CMV pp65_495-503_ (NLVPMVATV) or Melan-A/Mart-1_26-35_ (ELAGIGILTV) peptide epitopes. 2x10^6^ PBMC were stained with the amine-reactive viability dye ViViD, then stained with tetramer (1μg in minimal staining volume) ± pre-treatment with dasatinib for 30 min at 37 °C. Cells were then stained with cell surface markers as described in the Materials and methods; a dump channel was used to exclude dead cells, CD14^+^ and CD19^+^ cells from the analysis. Boolean gating was carried out to exclude aggregates. Data were acquired with a BD LSR II flow cytometer and analyzed using FlowJo software.

The above findings suggest that dasatinib treatment might enable the identification of low avidity antigen-specific CD8^+^ T-cells directly *ex vivo* that cannot be ‘seen’ in the absence of the drug. To test this idea, we first examined the staining of CTL lines in the presence or absence of 50 nM dasatinib. Staining improvements were observed in three different CTL lines raised against the Melan-A/Mart-1_26-35_ epitope (ELAGIGILTV) and three different CTL lines stimulated with the influenza matrix M1_58-66_ epitope (GILGFVFTL), all derived from HLA A2^+^ individuals. Representative data are shown in Fig. 4A. In all cases, dasatinib treatment substantially enhanced the staining intensity of cognate CD8^+^ T-cells without concomitant increases in the tetramer-negative population. In accordance with the results above, CD8^+^ T-cells that stained poorly with pMHCI tetramer exhibited the greatest benefit from dasatinib treatment. The staining intensity of all cognate CD8^+^ T-cells increased by at least 2-fold after dasatinib treatment, but T-cells that bound tetramer weakly exhibited increases of > 20-fold in their fluorescence intensity. In many cases, larger populations of cells that stained with the corresponding pMHCI tetramer were detected after dasatinib treatment. This increase in tetramer^+^ cells after dasatinib treatment likely reflects the combined effects of a lower detection threshold in terms of TCR/pMHCI affinity and the fact that dasatinib reduces pMHCI tetramer-induced cell death (Fig. 3). Subsequently, we examined whether dasatinib could enhance pMHCI tetramer staining of cognate CD8^+^ T-cells in direct *ex vivo* PBMC samples and enable the detection of antigen-specific CD8^+^ T-cells that are 'invisible' with routine staining procedures. Indeed, a substantial increase in both pMHCI staining intensity and the percentage of antigen-specific CD8^+^ T-cells was observed at both 4 °C and 37 °C in PBMC samples stained with HLA A2 tetramers specific for antigens derived from CMV, EBV and Melan A (Fig. 4B).

### 3.5 pMHCI staining of functional autoimmune CTL following dasatinib treatment

**Fig. 5:**
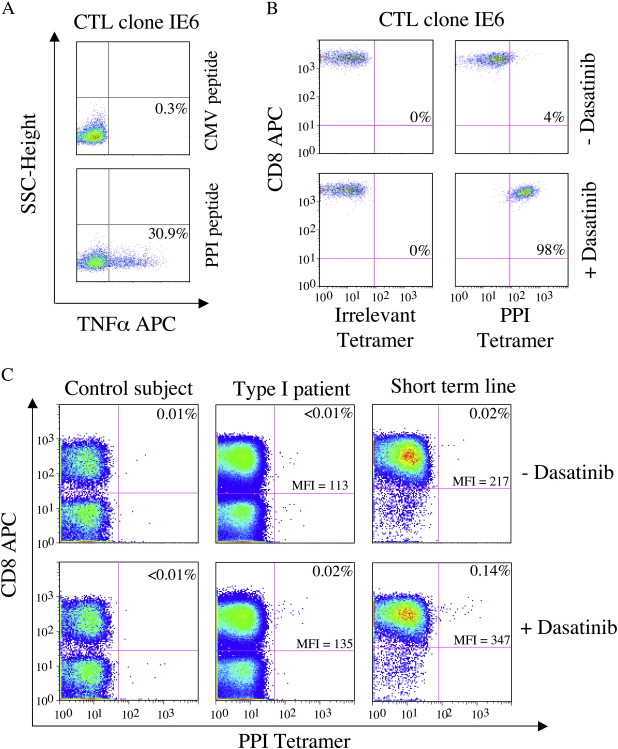
Dasatinib allows detection of autoreactive CTL. A. CTL clone IE6, specific for the HLA A2-restricted epitope PPI_15-24_, was activated with either CMV pp65_495-503_ or PPI_15-24_ peptide for 6 h at 37 °C and then assayed for TNFα production by intracellular cytokine staining as detailed in the Materials and methods. B. Staining of CTL clone IE6 with either an irrelevant or HLA A2/PPI_15-24_ tetramer ± pre-treatment with 50 nM dasatinib for 30 min at 37 °C. C. Representative stainings with HLA A2/PPI tetramer ± pre-treatment with 50 nM dasatinib for 30 min at 37 °C. Left panels: control subject PBMCs; middle panels: type I diabetic patient PBMCs; right panels: a short-term line expanded by one round of peptide stimulation from a type 1 diabetic patient.

We next examined pMHCI tetramer staining of IE6, a preproinsulin (PPI_15-24_)-specific HLA A2- restricted autoreactive CTL clone isolated from a patient with type 1 diabetes. This CTL clone produces TNFα, IFNγ and MIP1*β* on stimulation with target cells pulsed with cognate PPI-derived peptide antigen (Fig. 5A), and does not stain with cognate pMHCI tetramer using conventional staining procedures (Fig. 5B). An identical result was obtained for 2D6, a different PPI-specific CTL clone isolated from a patient with type I diabetes (data not shown). Dasatinib treatment allowed both CTL clones to bind cognate tetramer without affecting staining with non-cognate tetramer (Fig. 5B & data not shown). In keeping with these findings, dasatinib treatment allowed the identification of a HLA A2/PPI_15-24_ tetramer-positive population directly *ex vivo* from a type I diabetic patient, consistent with a corresponding IFNγ ELISpot response to PPI_15-24_ peptide of 13 responder cells per 10^6^ PBMCs (Fig. 5C & data not shown). Dasatinib did not increase direct *ex vivo* HLA A2/PPI_15-24_ tetramer staining in healthy HLA A2-matched control subjects (Fig. 5C). A seven-fold increase in the percentage of autoreactive CTL was observed when short-term lines expanded from two type I diabetic patients were stained in the presence of dasatinib (Fig. 5C & data not shown). Thus, dasatinib treatment allows the detection of functional autoreactive CTL that are otherwise undetectable with standard staining conditions.

### 3.6 How does dasatinib exert its beneficial effects on pMHC tetramer staining?

**Fig. 6:**
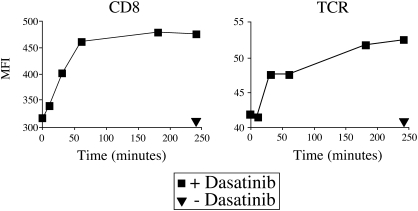
Dasatinib results in a time dependent increase in TCR and CD8 expression levels at the CTL cell surface. The ILA1 CTL clone was treated with PBS ± 50 nM dasatinib at 37 °C and 10^5^ CTL were removed from the medium at 0, 10, 30, 60, 180 and 250 min. CTL were subsequently stained with anti-CD8 FITC (clone SK1; BD, Pharmingen; left panel) or anti-TCR FITC (clone BMA 031; Serotec; right panel) for 30 min on ice, washed twice and resuspended in PBS. Data were acquired on a FACSCalibur flow cytometer (BD) and analyzed using FlowJo software.

Previous studies have demonstrated that incubation with Src kinase inhibitors results in enhanced TCR and CD8 expression at the cell surface (Luton et al., 1994; D'Oro et al., 1997). Consistent with these observations, we have recently demonstrated that increased levels of TCR and CD8 are seen at the cell surface following incubation with dasatinib for 4 h (Weichsel et al., 2008). Initially, therefore, we investigated this increase in TCR and CD8 levels as a possible mechanism for the observed effects on tetramer binding. The beneficial effects of dasatinib on pMHCI tetramer staining were observed within seconds of dasatinib treatment (Fig. 1D), whereas significant increases in TCR and CD8 levels were not observed until > 30 min (Fig. 6). Therefore, this time dependent accumulation of TCR and CD8 at the cell surface cannot explain the effects of dasatinib on tetramer binding.

**Fig. 7:**
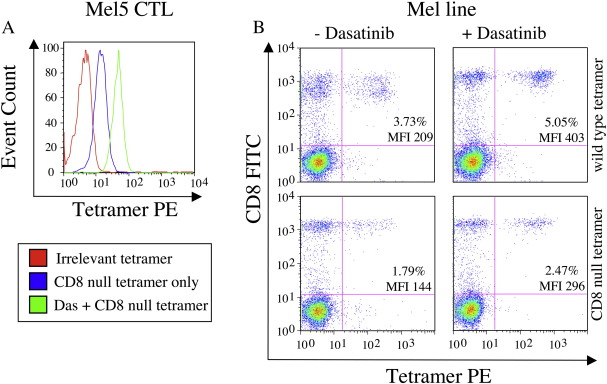
Beneficial effects of dasatinib are not CD8-mediated. A. Melc5 CTL were pre- treated with PBS ± 50 nM dasatinib for 30 min at 37 °C, then stained with HLA A2 DT227/8KA cognate tetramer for 20 min at 37 °C. After washing twice, data were acquired on a FACSCalibur flow cytometer (BD) and analyzed using FlowJo software. B. Staining of HLA A2-restricted CTL lines expanded from PBMC by one round of stimulation with the Melan-A/Mart-1_26-35_ peptide (ELAGIGILTV). Lines were stained with either wild type or CD8 null cognate tetramer ± pre-treatment with 50 nM dasatinib for 30 min at 37 °C.

We next investigated whether the mechanism of PKI action operates through TCR- or CD8-mediated effects. Dasatinib treatment enhanced pMHCI tetramer staining of the HLA A2-restricted ELAGIGILTV-specific Melc5 CTL clone and a CTL line raised against the Melan-A/Mart-1_26-35_ epitope (ELAGIGILTV) with both wildtype and CD8-null (DT227/8KA) tetramers (Fig. 7A&B), thereby demonstrating that PKIs can exert their effects in the absence of a pMHCI/CD8 interaction. Thus, consistent with effects on pMHCII tetramer binding (Fig. 1), dasatinib does not enhance pMHCI tetramer binding via CD8-mediated effects.

**Fig. 8:**
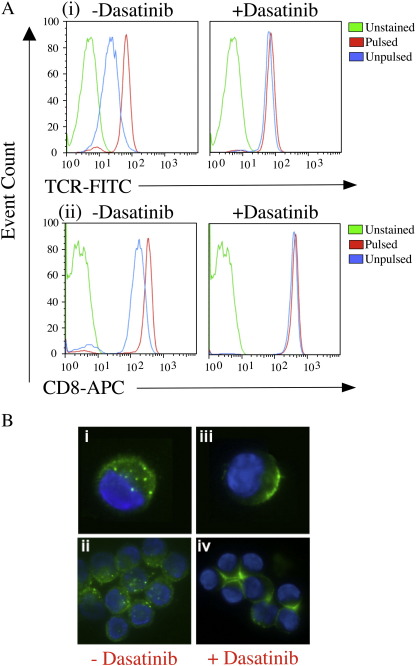
Dasatinib blocks antigen-induced TCR downregulation and tetramer internalization from the cell surface. A. Mel13 CTL were pre-treated with PBS ± 50 nM dasatinib and exposed to C1R-A2 B cells previously pulsed with 10^-6^M ELAGIGILTV peptide or medium alone for 4 h at 37 °C. Cells were subsequently stained with anti-TCR-FITC (clone BMA 031; Serotec) and anti-CD8-APC (clone RPA-T8; BD Pharmingen) mAbs for 30 min on ice, washed twice and resuspended in PBS. Data were acquired on a FACSCalibur flow cytometer (BD) and analyzed using FlowJo software. B. 10^5^ ILA1 CTL were pre-treated with PBS (i & ii) or PBS + 50 nM dasatinib (iii & iv) for 30 min at 37 °C, then stained with 20μg/ml HLA A2/ILAKFLHWL-Alexa488 tetramer for 15 min at 37 °C. Microscopy was performed as described in the Materials and methods.

**Fig. 9:**
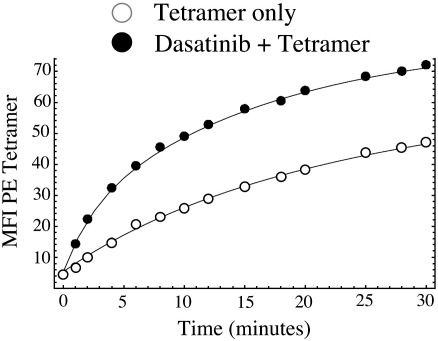
Dasatinib enhances pMHCI tetramer on-rate. Rate of HLA A2/hTERT_540-548_ (ILAKFLHWL) tetramer recruitment to the cell surface of clone ILA1 is substantially enhanced following treatment of CTL with 50 nM dasatinib for 30 min at 37 °C. Subsequent to treatment with dasatinib, on-rate experiments were performed and analyzed as described previously (Laugel et al., 2007). Curves represent the following rate estimates: fast rate 0.14/min, slow rate 0.04/min (tetramer only); fast rate 0.42/min, slow rate 0.06/min (dasatinib + tetramer).

TCR expression levels are not static and TCRs are constantly being down-regulated from the cell surface (Krangel, 1987). TCR internalization is thought to be mediated by three different mechanisms: (i) constitutive recycling of the TCR between intracellular compartments and the plasma membrane in resting cells by an unknown mechanism (Dietrich et al., 2002); (ii) protein kinase C activation (Minami et al., 1987; Dietrich et al., 2002); and, (iii) lck-mediated tyrosine phosphorylation following TCR ligation by specific pMHCI ligand. Dasatinib has been shown to target lck and therefore has the potential to inhibit the latter pathway of TCR down-regulation. Indeed, dasatinib treatment was found to block antigen-induced TCR downregulation from the CTL surface (Fig. 8A). pMHC tetramers are rapidly internalized under normal staining conditions (Whelan et al., 1999) and therefore we reasoned that dasatinib might exert its beneficial effects by blocking this process. To this end, fluorescence microscopy was performed in the presence and absence of dasatinib (Fig. 8B). HLA A2/ILAKFLHWL-Alexa488 tetramer capping and internalization was blocked in the presence of dasatinib and remained on the cell surface where it formed a ring that was visibly brighter than tetramer that had been internalized (Fig. 8B). Thus, by preventing TCR downregulation, PK inhibition acts to drive the system towards a higher number of surface TCRs and a higher number of potential productive engagements with pMHC tetramer. This has the effect of increasing tetramer on-rate, at least in pMHCI systems (Fig. 9).

## 4 Discussion

pMHC tetramer technology has revolutionized the study of antigen specific T-cells. However, one major limitation of this technique is that pMHCI, and most likely pMHCII, tetramer staining is dependent on a distinct TCR affinity threshold (Laugel et al., 2007). Consequently, pMHC tetramers fail to identify T-cells that express TCRs with low affinity for cognate antigen; such low affinity interactions characterize TCR/pMHCI binding in tumor-specific and autoreactive CD8^+^ T-cells (Cole et al., 2007). Here, we demonstrate that a short incubation with a reversible PKI such as dasatinib results in three major benefits in terms of pMHC tetramer staining. First, substantial improvements in pMHC tetramer staining intensity are observed. This effect applies to both CD4^+^ and CD8^+^ T-cells (Fig. 1). Indeed, the beneficial effects are so striking even at low pMHC tetramer concentrations that dasatinib treatment could be used to conserve reagent. Second, dasatinib treatment reduces tetramer-induced cell death that has been previously reported to be an issue with pMHCI tetramer staining protocols (Purbhoo et al., 2001; Xu et al., 2001; Guillaume et al., 2003; Cebecauer et al., 2005). Third, the benefits of dasatinib treatment are greater for TCR/pMHCI interactions of weak affinity and, as a result, dasatinib enhances the detection of low avidity CD8^+^ T-cells; this effect increases the number of CD8^+^ T-cells that can be detected directly *ex vivo*, particularly in the setting of tumor-specific and autoreactive CD8^+^ T-cell populations. Such effects are also likely to apply to CD4^+^ T-cells, which typically bind cognate pMHCII antigens with affinities lower than those reported for pMHCI systems (Cole et al., 2007).

Importantly, no increase in background staining was seen in any of the systems tested here (Figs. 1B, 1H, 5B & 5C). In fact the tetramer negative background was actually seen to decrease with dasatinib treatment in some staining experiments (Figs. 4C & 5C). Positive staining of PBMC in the presence of dasatinib only ever coincided with a positive ELISpot result to the relevant peptide. Dasatinib proved to be a particularly powerful tool in the detection of autoreactive CTL from type I diabetic patients. No increase in the PPI tetramer positive population was observed in healthy donors and indeed staining was only ever seen if a functional response to the preproinsulin peptide was evident. Our results suggest that the benefits of dasatinib treatment apply only to CTL that express TCR specific for the pMHCI tetramer in use and capable of recognizing cognate pMHCI (although function may be impaired in some low avidity CTL populations). This conclusion is further strengthened by the finding that the beneficial effects are TCR mediated and do not involve the CD8 coreceptor. Therefore dasatinib facilitates the specific TCR/pMHCI interaction rather than the non-specific pMHCI/CD8 interaction.

**Fig. 10:**
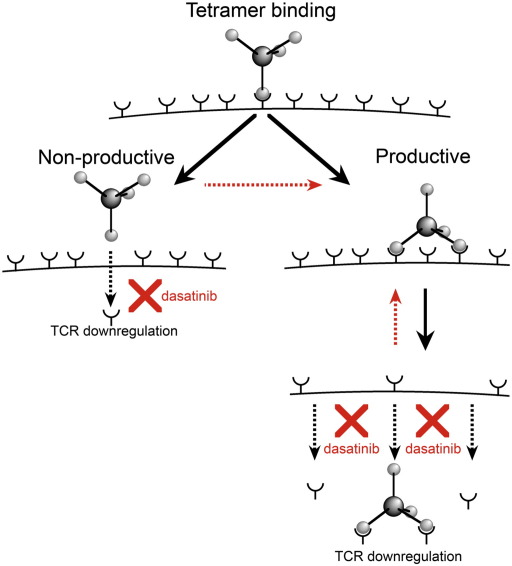
Dasatinib prevents down-regulation of ‘empty’ TCRs. Proposed model for the mechanism by which dasatinib enhances cognate tetramer staining. Dasatinib treatment prevents TCR and coreceptor down-regulation and maintains these receptors at the cell surface, thereby increasing molecular availability for further capture of pMHC tetramer from solution.

Dasatinib prevents TCR downregulation and tetramer internalization from the cell surface. How does this effect result in faster tetramer on-rates and the beneficial effects described above? When an individual pMHC molecule in a pMHC tetramer engages a cell surface TCR, this engagement can be either ‘productive’ or ‘non-productive’ in terms of capturing the tetramer from solution (Fig. 10). A productive engagement requires a second pMHC in the tetramer to bind a second TCR before the first pMHC dissociates. As a result, the main factor that determines whether a pMHCI tetramer exhibits stable binding is likely to be the duration of the primary monomeric TCR/pMHCI interaction. ‘Non-productive’ engagements are more likely to occur for low affinity ligands as they dissociate rapidly from cell surface TCR. Dasatinib would act to prevent TCR downregulation after ‘non-productive’ engagement thereby maintaining TCRs at the cell surface where they are available for future interactions with pMHCI. In addition, dasatinib treatment is likely to block the internalization of non-triggered TCRs that has been shown to occur at the same time as the internalization of pMHCI-engaged TCRs (Niedergang et al., 1997; San Jose et al., 2000). Dasatinib and other effective PKIs prevent TCR downregulation, acting to maintain surface TCRs and enabling a higher number of potential productive engagements with pMHC tetramer as indicated by the red arrows (Fig. 10). Increased TCR availability at the T cell surface would increase the likelihood of a ‘productive engagement’ for low affinity ligands. This effect would be less obvious for higher affinity ligands, where slow TCR/pMHCI off-rates already increase the likelihood that a second pMHCI arm can bind before dissociation of the first monomeric TCR/pMHCI interaction. The beneficial effects observed do not involve significant contribution from the CD8 coreceptor suggesting that CD8 availability at the T cell surface must be sufficient for pMHCI tetramer staining in the absence of dasatinib treatment.

In summary, we have demonstrated that a short incubation with reversible PKIs such as dasatinib substantially improves the staining intensity of cognate T-cells with pMHC tetramers and can expose concealed antigen-specific T-cells that bear low affinity TCRs. These benefits are restricted to cognate T-cells and are not accompanied by concomitant increases in background staining. As such, dasatinib has many potential uses; (i) enhancement of routine pMHCI tetramer staining in the lab, (ii) conservation of this valuable reagent as the amount required is significantly reduced in the presence of dasatinib, (iii) detection of low avidity CTL populations which will be of particular importance for researchers and clinicians studying diseases characterized by CTL of this type such as chronic viral infection, auto-immune and neoplastic disease. The beneficial effects of this reagent also extend to MHCII tetramers, with which staining is often very poor or not visible at all. We conclude, that this simple and universally applicable technique is likely to be beneficial in all studies of antigen-specific T-cells.

## Figures and Tables

**Table 1:** Affinity measurements of the interaction between the ILA1 TCR and hTERT_540-548_ pMHCI variants

LIGAND	8E	5Y	4L	Index	8Y	3G8T	3G
K_D_ ILA1 TCR Binding (μM)	> 500	242	117	36.6	22.6	4.04	3.7

Summary of the results obtained by nonlinear analysis of surface plasmon resonance binding equilibrium experiments as detailed in (Laugel et al., 2007) and (Melenhorst et al., 2008). K_D_ values were determined by analyzing the data in nonlinear curve fittings to the equation AB = B x AB_max_ / (K_D_ + B) assuming 1:1 Langmuir binding.
